# The repressive effect of miR-148a on Wnt/β-catenin signaling involved in Glabridin-induced anti-angiogenesis in human breast cancer cells

**DOI:** 10.1186/s12885-017-3298-1

**Published:** 2017-05-02

**Authors:** Juan Mu, Dongmei Zhu, Zhaoxia Shen, Shilong Ning, Yun Liu, Juan Chen, Yuan Li, Zhong Li

**Affiliations:** Department of Nutrition and Food Hygiene, The Key Laboratory of Modern Toxicology, Ministry of Education, School of Public Health, Nanjing Medical University, Nanjing, 211100 China

**Keywords:** Breast cancer, Angiogenesis, Glabridin, microRNA-148a, Wnt/β-catenin signaling

## Abstract

**Background:**

Glabridin (GLA), a major component extracted from licorice root, has anti-inflammatory and antioxidant activities, but few studies report its mechanism of inhibition of angiogenesis. This study was an extension of our previous work, which demonstrated that GLA suppressed angiogenesis in human breast cancer (MDA-MB-231 and Hs-578T) cells. Breast cancer is one of the most common malignant diseases in females worldwide, and the major cause of mortality is metastasis that is primarily attributed to angiogenesis. Thus, anti-angiogenesis has become a strategy for the treatment of breast cancer.

**Methods:**

Cell viability of different concentration treatment groups were detected by Cell Counting Kit-8 assay. The expression of several related genes in the Wnt1 signaling pathway in MDA-MB-231 and Hs-578T cells treated with GLA were measured at both the transcription and translation levels using quantitative real-time PCR analyses and western blotting. Immunofluorescence assay analyzed the nuclear translocation of β-catenin. The microRNA-inhibitor was used to knockdown microRNA-148a (miR-148a) expression. Angiogenic potentials of breast cancer cells were analyzed by enzyme-linked immunosorbent assay (ELISA) and tube formation in vitro.

**Results:**

GLA attenuated angiogenesis by the suppression of miR-148a-mediated Wnt/β-catenin signaling pathway in two human breast cancer cell lines (MDA-MB-231 and Hs-578T). GLA also upregulated the expression of miR-148a in a dose-dependent manner, miR-148a, which could directly target Wnt-3′-untranslated regions (UTRs), and decreased the expression of Wnt1, leading to β-catenin accumulation in the membranes from the cytoplasm and nucleus. Downregulation of miR-148a contributed to the reduction of GLA-induced suppression of the Wnt/β-catenin signaling pathway, the angiogenesis and vascular endothelial grow factor (VEGF) secretion.

**Conclusions:**

Our study identified a molecular mechanism of the GLA inhibition of angiogenesis through the Wnt/β-catenin signaling pathway via miR-148a, suggesting that GLA could serve as an adjuvant chemotherapeutic agent for breast cancer.

**Electronic supplementary material:**

The online version of this article (doi:10.1186/s12885-017-3298-1) contains supplementary material, which is available to authorized users.

## Background

Angiogenesis plays a crucial role in the pathogenesis of various solid tumors. During tumorigenesis, tumor cells induce an environment with an abundance of pro-angiogenic factors to facilitate the formation of blood vessels that can infiltrate solid tumors [1]. Breast cancer is a major health problem worldwide. The invasion and metastasis of tumor rely on its vascular supply. The new blood vessels play an important role in this process, in which cancer cells pass through the basement membrane barrier to relocate to remote organs [2]. Therefore, it is important to develop novel antiangiogenic agents to treat patients with these tumors.

The Wnt signaling has been suggested to be involved in cell proliferation, apoptosis, migration, stem cell maintenance, and differentiation in different organs [3]. It also contributes to the deterioration and development of human cancer [4]. Numerous studies have reported that the dysregulation of the Wnt/β-catenin pathway contributes to the regulation of VEGF expression, tumor angiogenesis and that VEGF is a novel target of the Wnt pathway [5, 6].

Emerging evidence has emphasized the role of microRNAs as novel molecular regulators in Wnt/β-catenin signaling during angiogenesis [7]. As the small noncoding RNAs, the microRNAs can regulate the expression of target genes by binding to their 3′-UTRs [8]. Take colorectal cancer cells for instance. miR-29b downregulates Wnt signaling, then reduces tumor cell medium-induced tube formation in endothelial cells [9]. In addition, miR-10a plays a suppressive role in the angiogenic activity of mouse umbilical vein endothelial cells by reducing the protein and transcriptional levels of β-catenin [10]. Recent studies report that miR-148a is a novel microRNA that directly binds to the Wnt1 3′-UTR, to inhibit the epithelial-mesenchymal transition and cancer stem cell (CSC)-like properties of hepatocellular carcinomas (HCCs) [11]. Other studies have clarified that miR-148a is significantly decreased in breast cancer cells associated with tumor angiogenesis, function as tumor suppressors to inhibit angiogenesis by targeting ERBB3 [12]. The potential tumor suppressors miR-148a and miR-152 are important for breast cancer cell proliferation, colony formation, and angiogenesis by targeting IGF-IR and IRS1 and inhibiting their downstream PI3K/AKT and MAPK/ERK signaling pathways [13]. Nevertheless, whether miR-148a can affect the angiogenesis via Wnt/β-catenin signaling in breast cancer remains largely unclear.

Licorice root is a kind of traditional Chinese medicine that has been accepted for menopausal symptoms, coughing and fever [14, 15]. Glabridin(GLA), an isoflavane compound in licorice roots, has attracted extensive attention because of its various biological properties, including the anti-proliferative [16], anti-stem cell-like [17], and anti-cancer activities [18]. The results of a previous research had shown that GLA suppressed angiogenesis through FAK/Rho signaling in human non–small cell lung cancer A549 cells and human breast cancer MDA-MB-231 cells [19, 20]. Our previous data also detected that repressed NF-kB/IL-6/STAT-3 signal pathway might be responsible for the inhibition of GLA on the angiogenic ability of human breast cancer MDA-MB-231 cells [21]. Recent studies suggest GLA can upregulate miR-148a, suppress the activation of TGF-β/SMAD2 signaling, and attenuate CSC-like functions in HCC and breast cancer cells [17, 22]. In the following study, we hypothesized that in breast cancer cells, GLA partially inhibited angiogenesis through the Wnt/β-catenin signaling pathway and that miR-148a was involved in this process. MDA-MB-231 and Hs-578 T cells were used to verify these hypotheses and further reveal the potentially molecular mechanisms of GLA’s action.

## Methods

### Cell culture and reagents

MDA-MB-231 and HS 578 T cells were purchased from the American Type Culture Collection (ATCC, Rockville, MD, USA). Briefly, MDA-MB-231 and HS 578 T cells were maintained in L-15 medium (Life Technologies/Gibco, Grand Island, NY, USA) and Dulbecco’s modified Eagle’s medium (DMEM; Life Technologies/Gibco), respectively, supplemented with 10% fetal bovine serum (FBS, Life Technologies/Gibco) and 1% antibiotics (100 U/ml penicillin and 100 mg/ml streptomycin). MDA-MB-231 cells were grown at 37 °C in a humidified incubator without CO_2_, while Hs-578 T cells were grown at 37 °C in an incubator with 95% air and 5% CO_2_. GLA (99.0% purity) was obtained from Sigma-Aldrich (St. Louis, MO, USA). Only reagents of analytical grade or the highest grade were used in the present study.

### Cell vitality

Cell Counting Kit-8 (CCK8) from Dojindo Molecular Technologies (Kumamoto, Japan) was utilized to determine the cell vitality. MDA-MB-231 cells (2 × 10^3^) were seeded into a 96-well plate and grown for 24 h. Next, cells were administrated with GLA in different concentrations (0, 10, or 20 μM) for another 24 h or 48 h. After washing three times using sterile phosphate-buffered saline (PBS), cells were incubated with CCK-8 for 4 h. A Bio-Rad multi-well plate reader was used for detecting the absorbance at 450 nm.

### Determination of angiogenic potentials

VEGF secretion and tube formation were used to determine the angiogenesis of those breast cancer cells. In brief, after treatment as described above, we collected the cell culture medium. After purifying by centrifugation, the supernatant samples were stored at −80 °C. The VEGF protein secreted from cells was quantified utilizing a standard recombinant human VEGF protein (R&D Systems, Minneapolis, MN, USA) and the commercial human VEGF Quantikine kit (R&D Systems). The procedure was performed based on the manufacturer’s instructions. Human umbilical vein endothelial cells (HUVECs) were cultured for the tube formation assay. HUVECs were grown in RPMI-1640 (Life Technologies/Gibco) at 37 °C with 5% CO2 and were seeded into a 48-well Multiwell™ plate (BD Biosciences, San Jose, CA, USA) at the equal density of 5 × 10^4^ cells per well. After culture in the conditioned media as indicated previously for 6 h, the number of tube branches was counted in every well using an Olympus light microscope (Tokyo, Japan).

### Quantitative real-time polymerase chain reaction (qRT-PCR)

A standard method of TRIzol® from Invitrogen (Carlsbad, CA, USA) was adopted for RNA extraction in human breast cancer cells. After quantification, cDNA synthesis was performed using total RNA (2 μg) and AMV Reverse Transcriptase (Promega, Madison, WI, USA) for the measurement of mRNAs. While for the determination of miRNAs, the miRNAs-specific stem-loop RT primers, 2-μg total RNA as well as MMLV reverse transcriptase (Promega) were utilized for the reverse transcription. All primer sequences are shown in Additional file 1: Table S1. The amplification of cDNA was carried out in a real-time PCR machine (ABI 7300, Applied Biosystems by Life Technologies, Grand Island, NY, USA) with MaximaTM SYBR Green/ROX qPCR Master Mix (Fermentas, Waltham, MA, USA). Relative gene expression was determined by taking the expression ratio of the gene of interest to Glyceraldehyde-3-phosphate dehydrogenase (GAPDH). While for the detection of miR-148a, U6 snRNA was regarded as an internal control to normalize expression. Melting curve analysis was used to evaluate the PCR reaction and a comparative threshold cycle (Ct) method using the formula 2^-(ΔΔCt)^ was adopted to determine the fold changes of each gene expression.

### Western blotting

The cells were homogenized in RIPA buffer containing 1 mM PMSF protease inhibitor (Beyotime Biotechnology, Shanghai, China). Cell lysis samples were heated to 100 °C for 10 min and separated by sodium dodecyl sulfate polyacrylamide gel electrophoresis (SDS-PAGE, Beyotime Biotechnology). After transfer onto polyvinylidene fluoride membranes (PVDF; Millipore, Billerica, USA), the membranes were blocked in 5% bovine serum albumin (BSA) at room temperature for 1 h. The primary antibodies were prepared at a dilution of 1:500. After incubation with different primary antibodies (anti-β-catenin, anti-Wnt, and anti- non-phospho (active) β-catenin, Cell Signaling Technology, Beverly, MA, USA; anti-VEGF, Beyotime) at 4 °C overnight, the membranes were washing for 3 times. Next, the membranes were treated with horseradish peroxidase-conjugated secondary antibodies (Beyotime, dilution: 1:1000) at room temperature for 1 h. Then the membranes were scanned after pretreating with enhanced chemiluminescence (Cell Signaling Technology). The densitometry values of bands were quantified with an Eagle Eye II imaging system (Stratagene, La Jolla, CA, USA). GAPDH (Sigma–Aldrich) at a dilution of 1:1000 was utilized for normalizing the protein loading.

### Immunofluorescence

Methyl alcohol was used for fixing the treated cells for 10 min, then sealed with 1% sheep serum. The cells were then washed using 1× tween-buffered phosphate-buffered saline (PBST) solution (Beyotime) and incubated for 1 h with anti-β-catenin (1:400) monoclonal antibody at room temperature. Cells were washed five times by 1× PBST and β-catenin was performed following incubation with Alexa Fluor 555 (1:100, Beyotime) for 1 h at room temperature. And 4′, 6-diamidino-2-phenylindole (DAPI; Sigma) was used for 15 min to stain nuclei. An Olympus confocal scanning microscope (Tokyo, Japan) was used for observing and photographing the immunofluorescence signal.

### MicroRNA transfection

Briefly, breast cancer cells were seeded in six-well plates at a density of 1 × 10^5^ per well. After 48 h, anti-miR-negative control and anti-miR-148a (RiBoBio Guangzhou, China) were transfected in cells at 50 nM using Lipofectamine® 2000 (Invitrogen) following the standard protocol. After 12 h of transfection, the culture medium was replaced by fresh DMEM containing 10% FBS (Gibco) for another 24 h before further experiments. The information of miRNA inhibitors used in this study is shown in Additional file 2: Table S2.

### Statistical analysis

All data were presented as mean ± standard deviation (SD). Statistical analyses consisted of Student’s t-test, and one-way analysis of variance followed by post-hoc tests (Dunnett’s t-test) using Graphpad Prism 5 (Graphpad Software, Inc., La Jolla, CA, USA). Differences considered statistically significant only when *p*-values were less than 0.05.

## Results

### GLA reduces angiogenic capacity in MDA-MB-231 cells

**Fig. 1:**
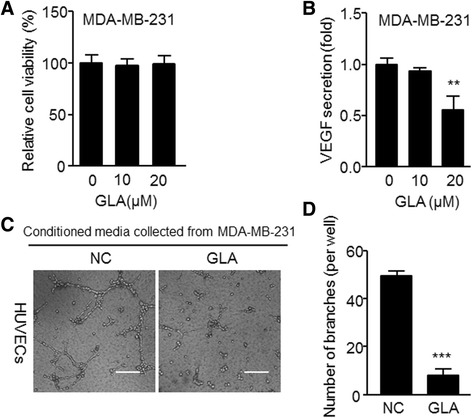
GLA reduces the angiogenic capacity in MDA-MB-231 cells. **a** MDA-MB-231 cells were exposed to 10 or 20 μM GLA for 48 h, using a Cell Counting Kit-8 assay of the cell vitalities, The percentage of cell viability was calculated via comparing with non-treated cells (mean ± SD, *n* = 3). Cells were then exposed to 10 or 20 μM GLA for 48 h, and conditioned media was collected. **b** The ELISA was used to detect the effects of GLA on VEGF secretion (mean ± SD, *n* = 3). MDA-MB-231 cells were pretreated with 0 or 20 μM GLA for 48 h, then the previous media was removed, and cells were washed with 1× PBS to replace fresh media with 1% serum for 24 h. The conditioned media was collected and incubated in (**c**) tube formation assays of the angiogenic capacity in MDA-MB-231 cells, HUVECs were exposed to the conditioned mediums collected as described in (**b**) for 6 h. **d** Quantitative analyses of the tube numbers, the total number of formed tube branches in each well was counted under the light microscope (mean ± SD, *n* = 5); ^**^ *P* < 0.01 and ^***^ *P* < 0.001 compared with cells treated without GLA

To assess the effects of GLA on cell viability, MDA-MB-231 breast cancer cells were treated with GLA at concentrations of 10 or 20 μM for 48 h and then analyzed by the Cell Counting Kit-8 assay. As shown (Fig. 1a), GLA did not significantly affect the viability of MDA-MB-231 cells compared with control cells. After incubation with GLA (10 μM or 20 μM) for 48 h (Fig. 1b and Additional file 1: Table S1A), the secretion of VEGF in breast cancer cells was markedly decreased after treatment with 20 μM GLA. Thus, this concentration was used in the following experiments. The tube formation assay was used to determine the angiogenic abilities of HUVECs when they were treated by conditioned media. MDA-MB-231 cells were administrated with GLA, after 48 h, the culture medium was substituted, and new fresh L-15 medium was added. After 24 h, the conditioned media from different groups were collected to treat HUVECs. The number of tubes decreased when compared with HUVECs incubated in media collected from controls (Fig. 1c–d). The results suggested that GLA attenuated the angiogenic ability of HUVECs incubated with MDA-MB-231, and this phenomenon was associated with the inhibition of VEGF secretion.

### GLA blocks Wnt/β-catenin signaling in MDA-MB-231 cells

**Fig. 2:**
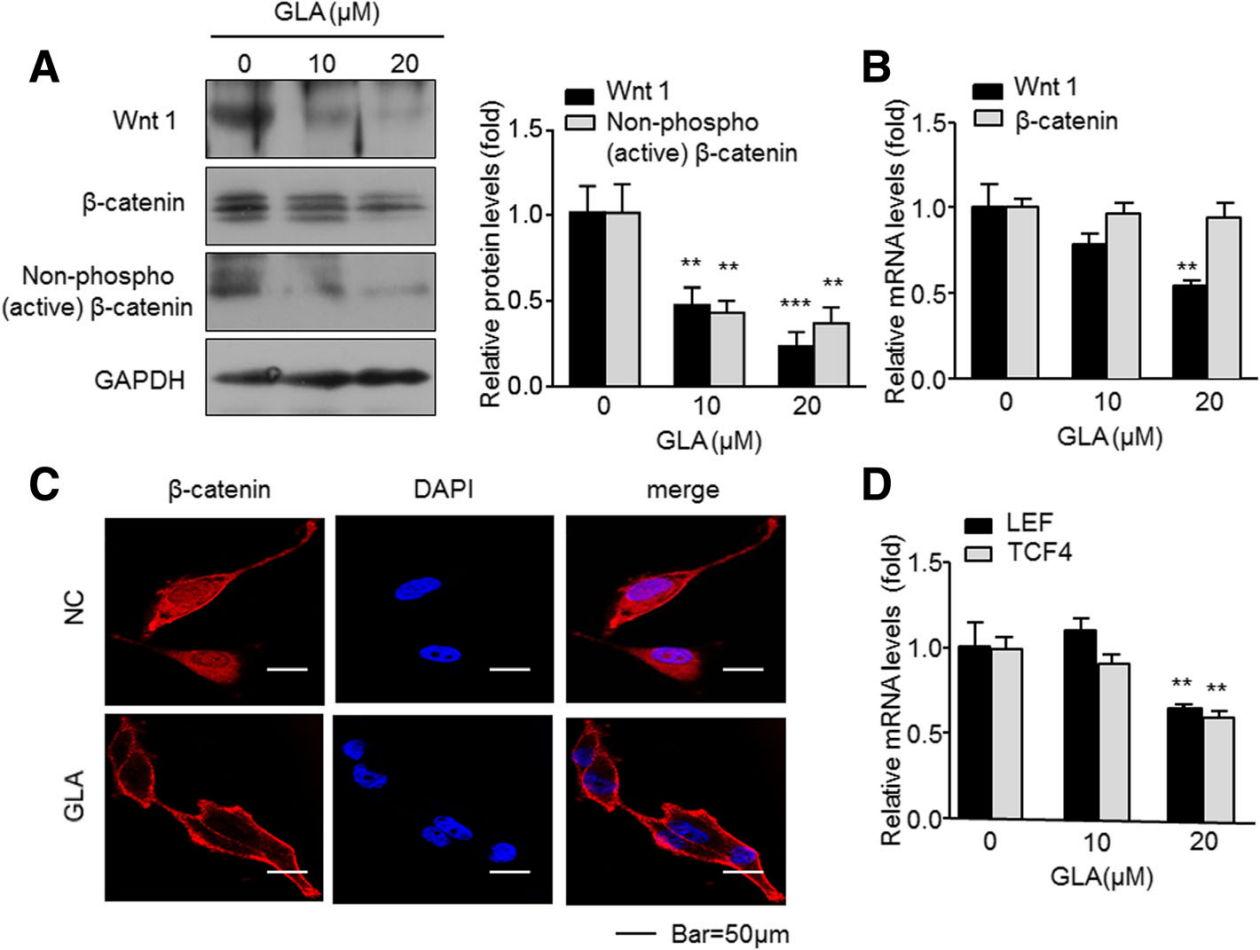
GLA blocks Wnt/β-catenin signaling in MDA-MB-231 cells. MDA-MB-231 cells were exposed to 10 or 20 μM GLA for 48 h. **a** Western blot analyses relative protein levels of Wnt1, non-phospho (active) β-catenin, and β-catenin. **b** Expression of Wnt1 and β-catenin (mRNAs) were analyzed by the quantitative real-time polymerase chain reaction (qRT-PCR) (mean ± SD, *n* = 3). **c** Immunofluorescence assay analyses the nuclear translocation of β-catenin. **d** Expression of TCF/LEF4 (mRNAs) analyzed by qRT-PCR (mean ± SD, *n* = 3). ^**^ *P* < 0.01 and ^***^ *P* < 0.001 compared with the control cells

Once Wnt was activated, accumulated β-catenin in cytoplasm and membranes translocated into the nucleus, and then modulated the transcription of the molecules of its downstream [23]. After 48 h treatment with GLA (10 or 20 μM), 20 μM GLA was shown to significantly decrease the protein expression of Wnt1 and non-phospho (active) β-catenin, as well as the mRNA expression of Wnt1 (Fig. 2a and b). Nevertheless, no changes were found in β-catenin mRNA and protein levels. So we further studied the localization of β-catenin. In MDA-MB-231 cells, β-catenin was mainly distributed in the nucleus and cytoplasm. But we found it began to return to cytosolic membranes when treated with 20 μM GLA (Fig. 2c). It has been demonstrated that the transcription factor lymphoid enhancer factor/T-cell factor 4 (LEF/TCF4) is an important downstream molecule of Wnt signaling pathway [24]. The transcriptional factors LEF/TCF4 can carry the upstream signal molecule β-catenin into the nucleus and activate Wnt pathways downstream target genes: *c-myc*, *cyclin D1*, *VEGF* and so on [25]. Here, we treated the MDA-MB-231cells with 20 μM GLA. As shown in Fig. 2d, GLA inhibited the mRNA expression of LEF/TCF4, suggesting that GLA could potentially suppress activation of transcriptional factors LEF/TCF4. Collectively, these data indicated that Wnt/β-catenin signaling can be blocked by GLA in human breast cancer cells.

### miR-148a interferes in the Wnt/β-catenin signaling in GLA-treated MDA-MB-231 cells

**Fig. 3:**
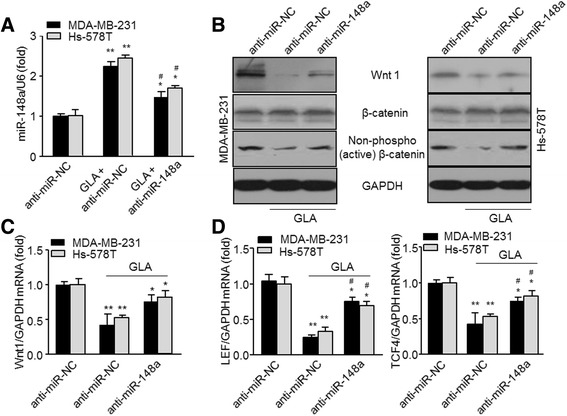
GLA attenuates the expression/activation of Wnt/β-catenin of breast cancer cells through miR-148a. **a**-**d** MDA-MB-231 or Hs-578 T cells were pre-transfected by anti-miR-negative control or anti-miR-148a for 12 h, and then treated with 20 μM GLA for 48 h. **a** qRT-PCR analyses of miR-148a (mean ± SD, *n* = 3). **b** Western blot analysis relative protein levels of Wnt1 and non-phospho (active) β-catenin. **c**–**d** Expression of Wnt1 and TCF/LEF were analyzed by qRT-PCR (mean ± SD, *n* = 3). ^*^ *P* < 0.05 and ^**^ *P* < 0.01 compared with the anti-miR-negative control. ^#^ *P* < 0.05 compared with the cells treated with GLA and anti-miR-negative control

A previous study suggested that miR-148a negatively regulated the epithelial to mesenchymal transition (EMT) and CSC-like properties of HCC by directly targeting Wnt1 [11]. In the present study, GLA increased the expression of miR-148a in breast cancer cells when exposed to 20 μM GLA for 48 h (Additional file 3: Figure S1B). Subsequently, we explored whether miR-148a could affect Wnt/β-catenin signaling under the treatment of GLA. After transfection with anti-miR-negative control or anti-miR-148a for 12 h, the efficiency of gene transfection in MDA-MB-231 or Hs-578 T cells was assayed (Additional file 3: Figure S1C). The transfected cells were maintained for 48 in culture medium with or without GLA (20 μM). After miR-148a knockdown, the downregulated protein level of Wnt1, non-phospho (active) β-catenin (Fig. 3b) and mRNA expression of Wnt1, LEF/TCF4 (Fig. 3c and d) induced by GLA were significantly further decreased, suggesting that GLA blocked the Wnt/β-catenin signal pathway through miR-148a.

### Functions of miR-148a in GLA-induced anti-angiogenesis in breast cancer cell

**Fig. 4:**
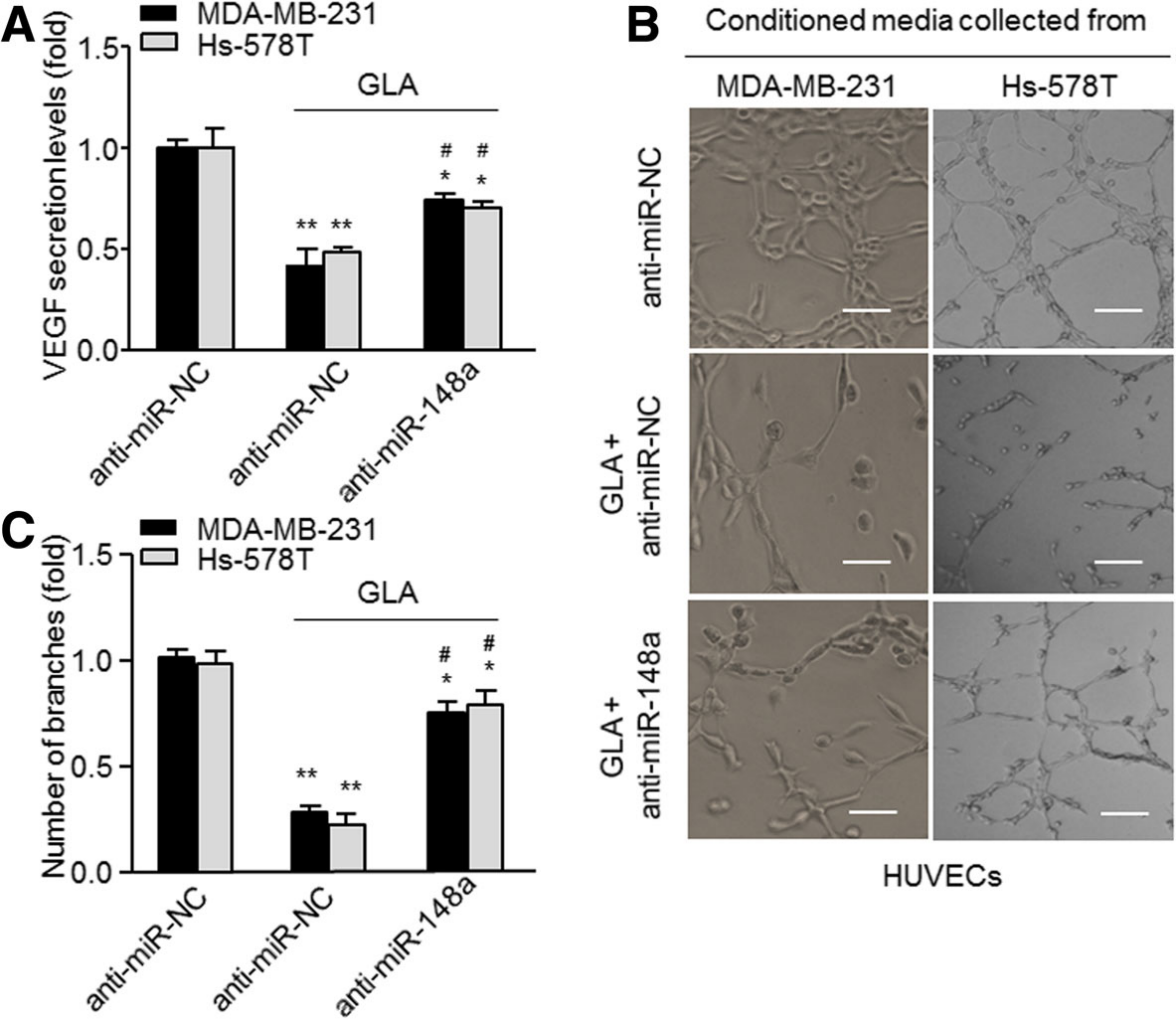
Functions of miR-148a in GLA-induced anti-angiogenesis. Cells were treated as described in Fig. 3, and the conditioned media were collected. **a** The ELISA was used to detect VEGF secretion (mean ± SD, *n* = 3). The previous media with anti-miRNAs and GLA were removed, and the cells were washed with 1× PBS and replaced with fresh media with 1% serum for 24 h, and the conditioned media was collected. **b** HUVECs were exposed to the conditioned mediums collected as described in (**a**) for 6 h. **c** Quantitative analyses of the tube numbers, the total number of formed tube branches in each well was counted under the light microscope (mean ± SD, *n* = 5). ^*^ *P* < 0.05 and ^**^ *P* < 0.01 compared with the anti-miR-negative control. ^#^ *P* < 0.05 compared with the cells treated with GLA and anti-miR-negative control

Based on our results, we hypothesized that the attenuation of Wnt/β-catenin signaling by miR-148a is involved in GLA-induced anti-angiogenesis in breast cancer cells. To test this hypothesis, we treated miR-148a knockdown MDA-MB-231 and Hs-578 T cells with GLA to determine their angiogenic abilities. miR-148a knockdown resulted in the decrease of GLA-induced suppression of VEGF expression/secretion (Fig. 4a) and tube formation (Fig. 4b and c) in these cells.

To avoid the interference of GLA and serum on HUVECs, human cancer cells were pretreated by GLA, then the culture medium was substituted, and new fresh medium with 1% FBS was added. After 24 h treatment, the conditioned media was collected. We found the up-regulation of miR-148a, the suppression of Wnt/β-catenin, and the down-regulation of VEGF. Our results further indicated that miR-148a-mediated inhibition of the Wnt/β-catenin signal pathway might be involved in GLA induced suppression of angiogenesis, and reduction of VEGF secretion (Additional file 4: Figure S2).

## Discussion

GLA, a flavonoid extracts from the phytochemical licorice, has multiple biological activities [14], such as estrogen-like [26] and anti-inflammatory activities [27]. In addition, GLA can activate caspase-3, −8, and −9 to induce HL-60 cell apoptosis through the regulation of the p38 MAPK and JNK1/2 pathways [18]. GLA inhibits migration and invasion by transcriptional inhibition of MMP 9 through modulation of NF-κB and AP-1 activity in human liver cancer cells. Hsieh et al. showed that GLA inhibited the transcription factors NF-κB, activator protein 1 signaling pathways and phosphorylation of ERK, JNK and p38 MAPKs in human liver cancer cells [28]. Our previous studies confirmed that GLA inhibited the CSC-like properties through the TGF-β/SMAD signaling pathway in HCC and breast cancer cells [17, 22]. Recent studies have revealed that GLA contributes to the inhibition of invasion, migration, and angiogenesis of breast cancer cells and lung cancer cells via a FAK/Rho mediated signaling pathway [19, 20]. In our study, we addressed the role of GLA in angiogenesis and found that GLA decreased the number of tubes in HUVECs by decreasing the expression and secretion of VEGF in breast cancer cells. In summary, the above results suggest that the tumor angiogenesis of breast cancer cells can be alleviated by GLA.

In cancer cells, the aberrantly activated Wnt/β-catenin signaling is able to regulate diverse biological processes, such as cell motility, migration, differentiation, proliferation as well as survival [29]. Wnt signaling is activated when it binds to the corresponding receptor, frizzled protein. After translocation into the cell nucleus, β-catenin binds with the TCF/LEF family and forms the complexes, modulating the transcription of various target genes, including *cyclin D1*, *interleukin-8*, and *VEGF*, which is a critical proangiogenic factor [30, 31]. For lung cancer cells, the inhibition of angiogenesis resulted from downregulation of the Wnt/β-catenin signaling axis [32]. The present study demonstrated that GLA treatment lead to the downregulation of Wnt1 in MDA-MB-231 and Hs-578 T cells. Besides, β-catenin was mainly distributed in the nucleus and cytoplasm, but we found its accumulation in membranes after GLA treatment. These results suggest that in the breast cancer cells, GLA can block the activation of Wnt/β-catenin signaling.

MiR-148a is a member of the miR-148/152 family that is usually regulated by methylation of CpG islands [33]. Growing evidence suggests that miR-148a is poorly expressed in various tumors, indicating that miR-148a can serve as a biomarker for diagnosis and prognosis [34]. miR-148a can regulated various target genes and its corresponding pathways, which is related to cell proliferation [35], invasion and metastasis [36], and angiogenesis [12]. In breast cancer cells, miR-148a inhibits tumor angiogenesis via targeting IGF-IR and IRS1 and suppressing their downstream AKT and MAPK/ERK signaling pathways [13]. Here, we explored the expression changes of four target genes of miR-148a (*ERBB3*, *PKM2*, *IGF-IR* and *IRS1*) in MDA-MB-231 cells followed by GLA addiction and silencing of miR-148a in Additional file 5: Figure S3. MiR-148a also modulates angiogenesis by directly targeting the M2 isoform of pyruvate kinase in mammary tumor cells [37]. Previous studies have shown that Wnt1 was a direct target of miR-148a [11]; however, little is known concerning the association of miR-148a with Wnt/β-catenin signaling during angiogenesis, so we aimed to uncover whether miR-148a was involved in the blockage of Wnt/β-catenin signaling. We determined the role of miR-148a in breast cancer angiogenesis after transfecting anti-miR-148a into breast cancer cells and found that the decreased expression or secretion of VEGF was reversed, indicating that miR-148a had a negative effect on angiogenesis.

## Conclusion

In conclusion, our findings suggest that GLA could be a promising chemopreventive drug in various cancers. Many investigators including us try to reveal its potential molecular mechanisms. Based on the previous peoples’ studies, our results further demonstrated that GLA could inhibit the growth and progression of tumors via affecting multiple signaling pathways. Using breast cancer MDA-MB-231 and Hs-578 T cells, we found that GLA decreased the formation of blood vessels by blocking the Wnt/β-catenin signaling pathway, which, in turn, reduced the secretion of a proangiogenesis factor (VEGF). Besides, GLA contributed to the over-expression of miR-148a, which could directly target Wnt1, and promoted the localization in membranes in the cytoplasm and nucleus. Downregulation of miR-148a reversed GLA-induced intervention of the Wnt/β-catenin signal pathway, the angiogenesis, and VEGF secretion. Therefore, these results suggest a novel mechanism whereby GLA inhibits angiogenesis, which may provide promising strategies to alleviate breast cancer in the future.

## Additional files

Additional file 1: Table S1. Primers used in this study. (DOCX 17 kb)

Additional file 2: Table S2. miRNA inhibitors used in this study. (DOCX 16 kb)

Additional file 3: Figure S1. Hs-578 T cells were exposed to 0, 10 or 20 μM GLA for 48 h, and conditioned media was collected. (A) The ELISA was used to detect the effects of GLA on VEGF secretion (mean ± SD, *n* = 3). MDA-MB-231 or Hs-578Tcells were exposed to 0, 10 or 20 μM GLA for 48 h, (B) qRT-PCR analyses the mRNA level of miR-148a (mean ± SD, *n* = 3). The breast cancer cells transfected by anti-miR-negative control or anti-miR-148a for 12 h, (C) the efficiency of gene transfection was analysed by qRT-PCR (mean ± SD, *n* = 3); *P < 0.05, **P < 0.01 and ***P < 0.001 compared with the control cells. (DOCX 195 kb)

Additional file 4: Figure S2. MDA-MB-231 cells were pretreated with 0, or 20 μM GLA for 48 h, then the media was removed, the cells were washed with 1× PBS, followed by replacement with fresh media with 1% FBS for 24 h. (A)The expression of miR-148a was analyzed by qRT-PCR (mean ± SD, *n* = 3). (B-C) Western blot analyses the relative protein levels of Wnt1, β-catenin, and non-phospho (active) β-catenin (mean ± SD, *n* = 3). (D) The ELISA was used to detect the secretion of VEGF (mean ± SD, *n* = 3); **P < 0.01 and ***P < 0.001 compared with the control media or cells. (DOCX 322 kb)

Additional file 5: Figure S3. MDA-MB-231 cells were pre-transfected by anti-miR-negative control or anti-miR-148a for 12 h, and then treated with 20 μM GLA for 48 h. (A-D) qRT-PCR analyses in triplicate of the mRNA level of ERBB3, PKM2, IRS1, and IGF-IR (mean ± SD, *n* = 3). *P < 0.05, and **P < 0.01 compared with the anti-miR-negative control. #*P* < 0.05 compared with the cells treated with GLA and anti-miR-negative control. (DOCX 255 kb)

## Abbreviations

AP-1: Activator protein 1
CCK8: Cell Counting Kit-8 assay
CSC: Cancer stem cell
DAPI: 4′, 6-diamidino-2-phenylindole
DMEM: Dulbecco’s modified Eagle’s medium
ELISA: Enzyme-linked immunosorbent assay
FAK/Rho: Focal adhesion kinase/Ras homolog
FBS: Fetal bovine serum
GLA: Glabridin
HCCs: Hepatocellular carcinomas
HUVECs: Human umbilical vein endothelial cells
IGF-IR: Insulin-like growth factor- I receptor
IL-6: Interleukin- 6
IRS1: Insulin receptor substrate 1
JNK1/2: Jun N-terminal kinase
LEF/TCF4: T cell factor/lymphoid enhancer binding factor 4
MAPK: Mitogen-activated protein kinase
NF-κB: Nuclear factor-κB
PBST: Tween-buffered phosphate-buffered saline
PVDF: Polyvinylidene fluoride
qRT-PCR: Quantitative real-time polymerase chain reaction
SDS-PAGE: Sodium dodecyl sulfate polyacrylamide gel electrophoresis
STAT-3: Signal transducer and activator of transcription 3
TGF-β: Transforming growth factor-β
UTRs: Untranslated regions
VEGF: Vascular endothelial grow factor
