# Supplementary material for: The repressive effect of miR-148a on Wnt/β-catenin signaling involved in Glabridin-induced anti-angiogenesis in human breast cancer cells
Source: BMC Cancer. 2017 May 2;17:307. doi: 10.1186/s12885-017-3298-1 (PMC5414299; doi:10.1186/s12885-017-3298-1)
Supplement: Supplementary file 1 — Primers used in this study. (DOCX 17 kb) [file 12885_2017_3298_MOESM1_ESM.docx]

**Additional file 1. T****able S1. Primers used in this study**

| TCF4 | Forward  Reverse | 5'-CAGAAGGCAGAGCGTGAG-3'  5'-GAGAAGTGTTTATGTGGGTTTA-3' |
| --- | --- | --- |
| LEF | Forward  Reverse | 5'-AGACAATCACTGCCAACC-3'  5'-TCCATGAACTCCTCCACT-3' |
| Wnt1 | Forward  Reverse | 5'-ACGTAGCCTCCTCCACGAACCTGC -3'  5'- CGCATCTCGGAGAATACGGTCG -3' |
| β-catenin | Forward  Reverse | 5'- CGTGGACAATGGCTACTCAAGC -3'  5'- TCTGAGCTCGAGTCATTGCATAC -3' |
| ERBB3 | Forward  Reverse | 5'- CCAAGACCATCTGTGCTCCT -3'  5'- TTGTCAGGAGGACAGGCCCT -3' |
| IGF-IR | Forward  Reverse | 5'- AACCCCAAGACTGAGGTGTG -3'  5'- TGACATCTCTCCGCTTCCTT -3' |
| IRS1 | Forward  Reverse | 5'- GGAGTACATG AAGATGGACCTGG -3'  5'- CTGTTCGCAT GTCAGCATAGC -3' |
| PKM2 | Forward  Reverse | 5'- GCCATAATCGTCCTCACCAAGT -3'  5'- GCACGTGGGCGGTATCTG -3' |
| GAPDH | Forward  Reverse | 5'-GACCTGACCTGCCGTCTA-3'  5'-GGAGTGGGTGTCGCTGT-3' |
| miR-148a | RT | 5’-CTCAACTGGTGTCGTGGAGTCGGCA-ATTCAGTTGAGAGTCGGAG-3’ |
| U6 | RT | 5’-AAAATATGGAACGCTTCACG-3’ |
| miR-148a | Forward  Reverse | 5’-ACACTCCAGCTGGGAAAGTTCTGA-  GACACT-3’  5’-TGGTGTCGTGGAGTCG-3’ |
| U6 | Forward  Reverse | 5’-CGCTTCGGCAGCACATATACTAAAAT-TGGAAC-3’  5’-GCTTCACGAATTTGCGTGTCATCCTT-GC-3’ |
