# Supplementary material for: The repressive effect of miR-148a on Wnt/β-catenin signaling involved in Glabridin-induced anti-angiogenesis in human breast cancer cells
Source: BMC Cancer. 2017 May 2;17:307. doi: 10.1186/s12885-017-3298-1 (PMC5414299; doi:10.1186/s12885-017-3298-1)
Supplement: Supplementary file 2 — miRNA inhibitors used in this study. (DOCX 16 kb) [file 12885_2017_3298_MOESM2_ESM.docx]

**Additional file 2. miRNA inhibitors used in this study**

| Names | Web Link | Source | |
| --- | --- | --- | --- |
| anti-miR-148a | http://www.ribobio.com/sitecn/product_info.aspx?  id=235787 | RiBoBio | |
| anti-miR-negative control | http://www.ribobio.com/sitecn/product_info.aspx?  id=45 | | RiBoBio |
