# Supplementary material for: The repressive effect of miR-148a on Wnt/β-catenin signaling involved in Glabridin-induced anti-angiogenesis in human breast cancer cells
Source: BMC Cancer. 2017 May 2;17:307. doi: 10.1186/s12885-017-3298-1 (PMC5414299; doi:10.1186/s12885-017-3298-1)
Supplement: Supplementary file 3 — Hs-578 T cells were exposed to 0, 10 or 20 μM GLA for 48 h, and conditioned media was collected. (A) The ELISA was used to detect the effects of GLA on VEGF secretion (mean ± SD, n = 3). MDA-MB-231 or Hs-578Tcells were exposed to 0, 10 or 20 μM GLA for 48 h, (B) qRT-PCR analyses the mRNA level of miR-148a (mean ± SD, n = 3). The breast cancer cells transfected by anti-miR-negative control or anti-miR-148a for 12 h, (C) the efficiency of gene transfection was analysed by qRT-PCR (mean ± SD, n = 3); *P < 0.05, **P < 0.01 and ***P < 0.001 compared with the control cells. (DOCX 195 kb) [file 12885_2017_3298_MOESM3_ESM.docx]

**Additional file 3. Figure S1**

**
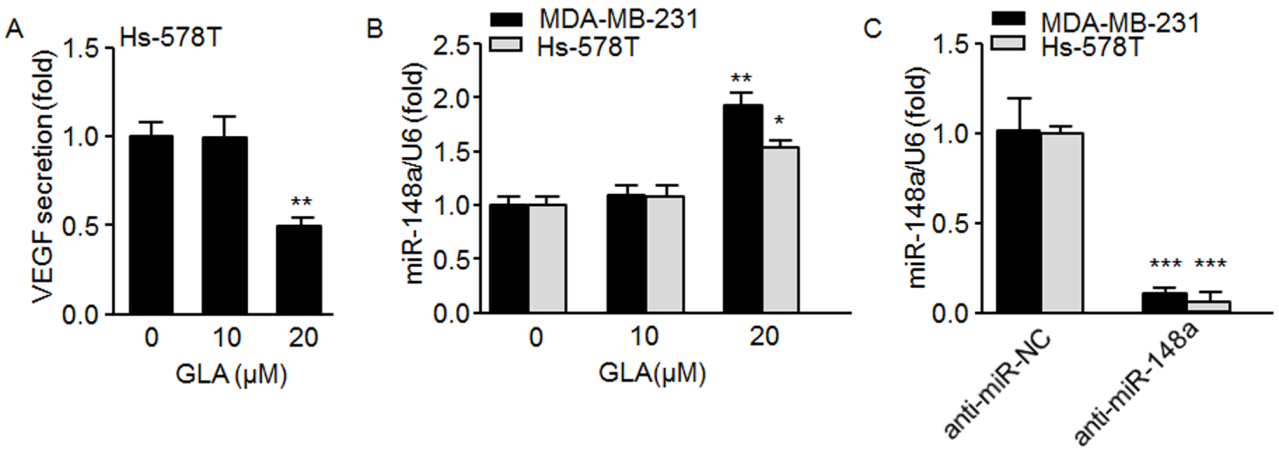
**

Figure S1. Hs-578T cells were exposed to 0, 10 or 20 μM GLA for 48 h, and conditioned media was collected. (A) The ELISA was used to detect the effects of GLA on VEGF secretion (mean ± SD, n = 3). MDA-MB-231 or Hs-578T cells were exposed to 0, 10 or 20 μM GLA for 48 h, (B) qRT-PCR analyses the mRNA level of miR-148a (mean ± SD, n = 3). The breast cancer cells transfected by anti-miR-negative control or anti-miR-148a for 12 h, (C) the efficiency of gene transfection was analysed by qRT-PCR (mean ± SD, n = 3); ^*^P < 0.05, ^**^P < 0.01 and ^***^P < 0.001 compared with the control cells.
