# Supplementary material for: The repressive effect of miR-148a on Wnt/β-catenin signaling involved in Glabridin-induced anti-angiogenesis in human breast cancer cells
Source: BMC Cancer. 2017 May 2;17:307. doi: 10.1186/s12885-017-3298-1 (PMC5414299; doi:10.1186/s12885-017-3298-1)
Supplement: Supplementary file 4 — MDA-MB-231 cells were pretreated with 0, or 20 μM GLA for 48 h, then the media was removed, the cells were washed with 1× PBS, followed by replacement with fresh media with 1% FBS for 24 h. (A)The expression of miR-148a was analyzed by qRT-PCR (mean ± SD, n = 3). (B-C) Western blot analyses the relative protein levels of Wnt1, β-catenin, and non-phospho (active) β-catenin (mean ± SD, n = 3). (D) The ELISA was used to detect the secretion of VEGF (mean ± SD, n = 3); **P < 0.01 and ***P < 0.001 compared with the control media or cells. (DOCX 322 kb) [file 12885_2017_3298_MOESM4_ESM.docx]

**Additional file 4. Figure S2**

**
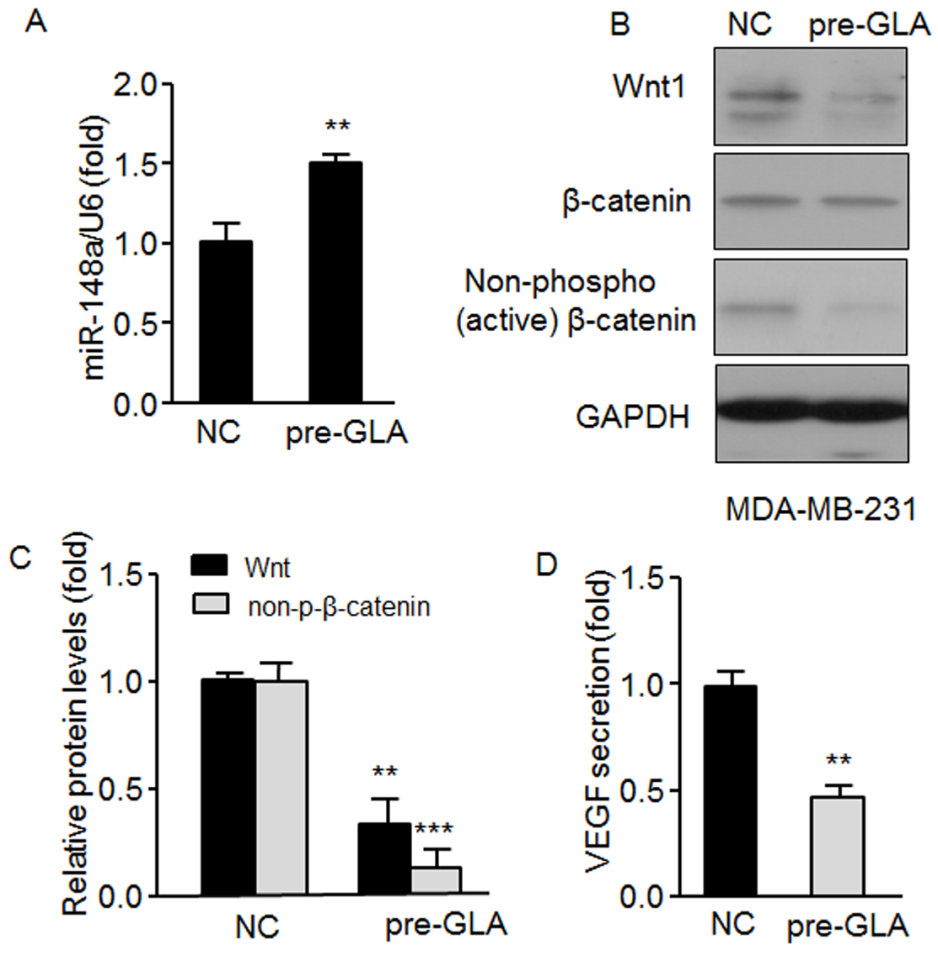
**

Figure S2. MDA-MB-231 cells were pretreated with 0, or 20 μM GLA for 48 h, then the media was removed, the cells were washed with 1× PBS, followed by replacement with fresh media with 1% FBS for 24 h. (A)The expression of miR-148a was analysed by qRT-PCR (mean ± SD, n = 3). (B-C) Western blot analyses the relative protein levels of Wnt1, β-catenin, and non-phospho (active) β-catenin (mean ± SD, n = 3). (D) The ELISA was used to detect the secretion of VEGF (mean ± SD, n = 3); ^**^P < 0.01 and ^***^P < 0.001 compared with the control media or cells.
