# Supplementary material for: The repressive effect of miR-148a on Wnt/β-catenin signaling involved in Glabridin-induced anti-angiogenesis in human breast cancer cells
Source: BMC Cancer. 2017 May 2;17:307. doi: 10.1186/s12885-017-3298-1 (PMC5414299; doi:10.1186/s12885-017-3298-1)
Supplement: Supplementary file 5 — MDA-MB-231 cells were pre-transfected by anti-miR-negative control or anti-miR-148a for 12 h, and then treated with 20 μM GLA for 48 h. (A-D) qRT-PCR analyses in triplicate of the mRNA level of ERBB3, PKM2, IRS1, and IGF-IR (mean ± SD, n = 3). *P < 0.05, and **P < 0.01 compared with the anti-miR-negative control. #P < 0.05 compared with the cells treated with GLA and anti-miR-negative control. (DOCX 255 kb) [file 12885_2017_3298_MOESM5_ESM.docx]

**Additional file 5. Figure S3**

**
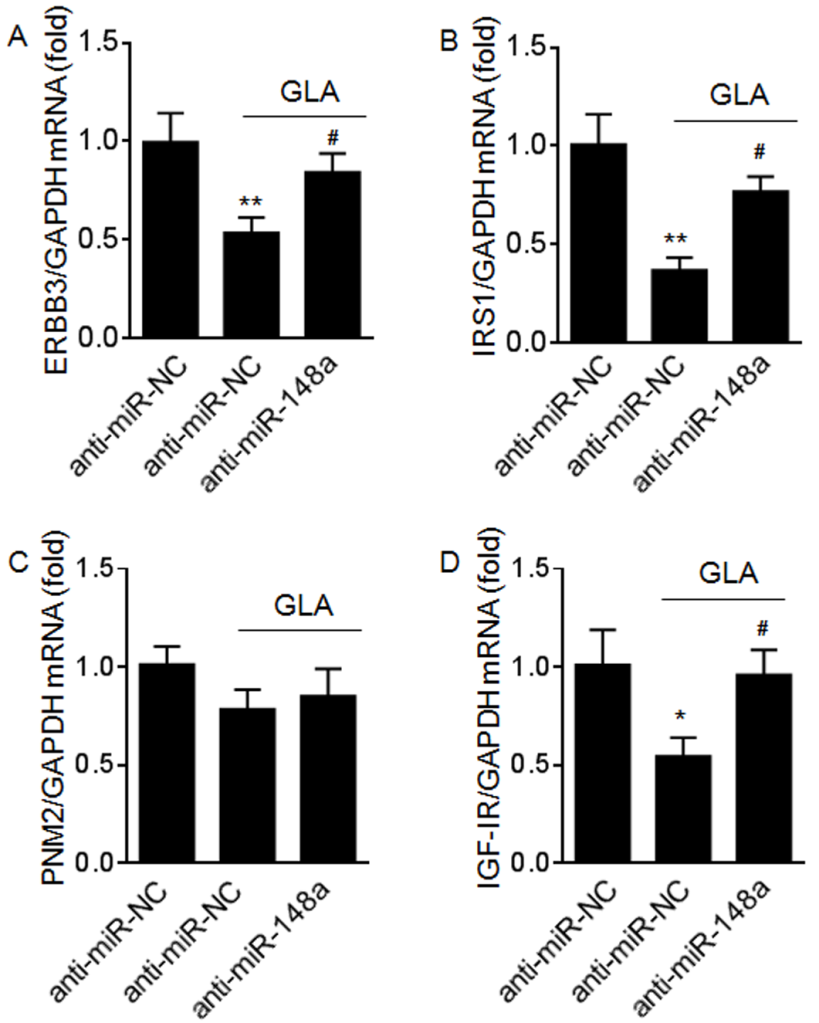
**

Figure S3. MDA-MB-231 cells were pre-transfected by anti-miR-negative control or anti-miR-148a for 12 h, and then treated with 20 μM GLA for 48 h. (A-D) qRT-PCR analyses in triplicate of the mRNA level of ERBB3, PKM2, IRS1, and IGF-IR (mean ± SD, n = 3). ^*^P < 0.05, and ^**^P < 0.01 compared with the anti-miR-negative control. ^#^P < 0.05 compared with the cells treated by GLA and anti-miR-negative control.
